# Engaging Through Awareness: Purpose-Driven Framework Development to Evaluate and Develop Future Business Strategies With Exponential Technologies Toward Healthcare Democratization

**DOI:** 10.3389/fpubh.2022.851380

**Published:** 2022-05-25

**Authors:** Beatrice Barbazzeni, Sultan Haider, Michael Friebe

**Affiliations:** ^1^ESF-GS ABINEP International Graduate School, Otto-von-Guericke-University, Magdeburg, Germany; ^2^Innovation Think Tank, Siemens Healthineers, Erlangen, Germany; ^3^INKA-HealthTec Innovation Laboratory, Medical Faculty, Otto-von-Guericke-University, Magdeburg, Germany; ^4^Department of Measurement and Electronics, AGH University of Science and Technology, Kraków, Poland

**Keywords:** innovation, disruptive technologies, Industry 4.0, healthcare democratization, revised education, artificial intelligence, patient-centric, Innovation Think Tank

## Abstract

Industry 4.0 and digital transformation will likely come with an era of changes for most manufacturers and tech industries, and even healthcare delivery will likely be affected. A few trends are already foreseeable such as an increased number of patients, advanced technologies, different health-related business models, increased costs, revised ethics, and regulatory procedures. Moreover, cybersecurity, digital invoices, price transparency, improving patient experience, management of big data, and the need for a revised education are challenges in response to digital transformation. Indeed, forward-looking innovation about exponential technologies and their effect on healthcare is now gaining momentum. Thus, we developed a framework, followed by an online survey, to investigate key areas, analyze and visualize future-oriented developments concerning technologies and innovative business models while attempting to translate visions into a strategy toward healthcare democratization. When forecasting the future of health in a short and long-term perspective, results showed that digital healthcare, data management, electronics, and sensors were the most common predictions, followed by artificial intelligence in clinical diagnostic and in which hospitals and homes would be the places of primary care. Shifting from a reactive to a proactive digital ecosystem, the focus on prevention, quality, and faster care accessibility are the novel value propositions toward democratization and digitalization of patient-centered services. Longevity will translate into increased neurodegenerative, chronic diseases, and mental illnesses, becoming severe issues for a future healthcare setup. Besides, data privacy, big data management, and novel regulatory procedures were considered as potential problems resulting from digital transformation. However, a revised education is needed to address these issues while preparing future health professionals. The “P4 of health”, a novel business model that is outcome-based oriented, awareness and acceptance of technologies to support public health, a different mindset that is proactive and future-oriented, and an interdisciplinary setting to merge clinical and technological advances would be key to a novel healthcare ecosystem. Lastly, based on the developed framework, we aim to conduct regular surveys to capture up-to-date technological trends, sustainable health-related business models, and interdependencies. The engagement of stakeholders through awareness and participation is the key to recognizing and improving healthcare needs and services.

## Introduction

Healthcare provision is an extremely complex system, in which providers, payers, and patients interact on multiple levels. The increasing rise of costs and patient expectations puts this care delivery system under continuous pressure, consequently preventing it from positively transforming. Increased global healthcare costs, limited democratized resources, unmet clinical needs, and increased longevity are just a few factors leading to a higher demand for better health services (1, 2). Indeed, demographic changes toward the aging population, affected by non-communicable chronic diseases (3–5), would demand continuous health monitoring and treatment access, prolonged hospitalizations, and patient management, as well as financing urgencies. To move toward improved healthcare delivery while at the same time promoting health-related developments (e.g., in research, management, manufacturing, care processes), novel value propositions supporting a preventing, predicting, personalized and participatory medicine (P4) (6–9) are highly needed.

For that to become reality several challenges have to be addressed and solved. Technology-enhanced medical expertise to improve patient care and management, better product distribution and access to quality manufacturers, and intellectual property connected to innovative solutions are just a few examples. Other obstacles to be solved are access to national health service data, ethical questions, regulatory procedures, a novel healthcare culture that embraces digital technologies, reducing the stress for healthcare workers. To many, it is not clear what health innovation is and how to pursue it (10). The 21st-century will represent a challenging era for the entire healthcare industry, in which several changes will be influencing and affecting any medical organization leading to a partial or complete rebuild of the health-related system and how it is functioning. Novel government regulations, the Covid-19 pandemic, technological advances, and improved patient care generate a different environment in which a revised approach to clinical practice is needed (11).

In the past decade, the United States' Academic Health Centers (AHCs) reported a decrease in the perceived value of patient assistance and a considerable reduction in payments to physicians. Consequently, this event has affected performance and productivity (e.g., patient care, research, and healthcare administration) (12). However, when speculating on the future of healthcare (13) several trends can be predicted such as the increasing number of patients (i.e., the ultimate consumers), disruptive technologies, a different business model to deliver healthcare, innovation driven by competition, increase of costs, uninsured individuals, decreased payment for providers, and lastly, the continuous need of updating and revising healthcare procedures and regulations (12). Hence, a multidisciplinary approach would be recommended to deliver more efficient and effective healthcare services (14, 15).

Furthermore, in the context of digital transformation and Industry 4.0 (16) more challenges need to be considered. To name a few, cybersecurity is one of the main concerns in healthcare to protect sensitive data. Indeed, the increasing implementation of digital health to record patient data highlights the need for more security. This factor is also reinforced by the growth of telehealth, which has seen fast growth and quick acceptance during the Covid-19 pandemic that forced the reduction of in-person interactions. Digital invoices and novel payment processing methods have been adapted to be patient-friendly facilitating online transactions in a timely manner. Price transparency is another challenge faced by patients and the healthcare system itself. More transparency would facilitate the patients' decisions in a self-paying health system when choosing a medical structure for performing care services. Even though digital health facilitates remote access and real-time health monitoring, to deliver efficient services, the improvement of patient experience is another aspect that providers need to face while sustaining the business.

A novel reimbursement model based on promoting prevention over treatment is proposed, in which cost reduction, patient-centric care, global payments, and shared savings are the relevant points. However, to match an effective payment model that guarantees higher service quality has to consider new metrics when evaluating performance and ROI. Lastly, the management of increased digital data, in a variety of formats, represents another potential issue that healthcare providers, payers, and patients have to solve. All of that requires the implementation of advanced technologies and data analytics to be able to manage and organize big data, supporting healthcare leaders toward a data-driven decision-making approach (11).

Advanced technologies are aimed to generate novel opportunities and value propositions, restructuring patient care and system management. In fact, the World Health Organization Health Innovation Group (WHIG) declares that health innovation “responds to unmet public health needs by creating new ways of thinking and learning” and “aims to add value in the form of improved efficiency, effectiveness, quality, sustainability and/or affordability” (17). For that novel therapies, surgical procedures, instruments, tests, revised education, and training programs, become a necessity. While digital transformation is highly connected with healthcare innovation enhancing patient care and experience, organizations additionally have to constantly deal with technologically advancing procedures and tools (18).

In response to global changes and challenges that are currently forcing healthcare to innovate and adapt, our aim was to develop a framework to identify relevant key areas, analyze and visualize future trends with respect to exponential technologies and innovative health-related business models. Moreover, the developed framework would represent a structure to visualize, understand and translate the investigated visions into a strategy toward healthcare democratization. Thus, to investigate technological and clinical developments about the future of health, we engaged stakeholders by conducting an online survey to help shape the development and business activities of a large medical equipment producer (19). Indeed, the online research survey, sent to graduate students (mainly health economics and biomedical engineering), employees, and employers in the medical sector, was created to explore respondents' personal vision about future applications in a short term (3–5 years approach) and long term (>10 years approach) perspective.

Furthermore, Innovation Think Tank (ITT) department of Siemens Healthineers (SHS), a global medical technology company that contracted this research questionnaire, runs co-creation projects at its global locations at SHS and several of its partner universities and hospitals (20).

ITT also organizes 8–10 times a year, a 4–15-day educational certification program with the goal to gain insights and create projects related to the future of health in a mid-term perspective based on the implementation of advanced medical technologies. Through “experiential learning training,” the interdisciplinary collaboration of innovative labs at the company and prestigious global research and education institutions is stimulated. Successively, some of these projects are subsequently further developed into a medical technology and disease pathway strategy. At the same time, these projects would also support further joint research while gaining insight toward outstanding and creative patient-centric solutions to raise healthcare innovation (14).

## Method

**Figure 1:**
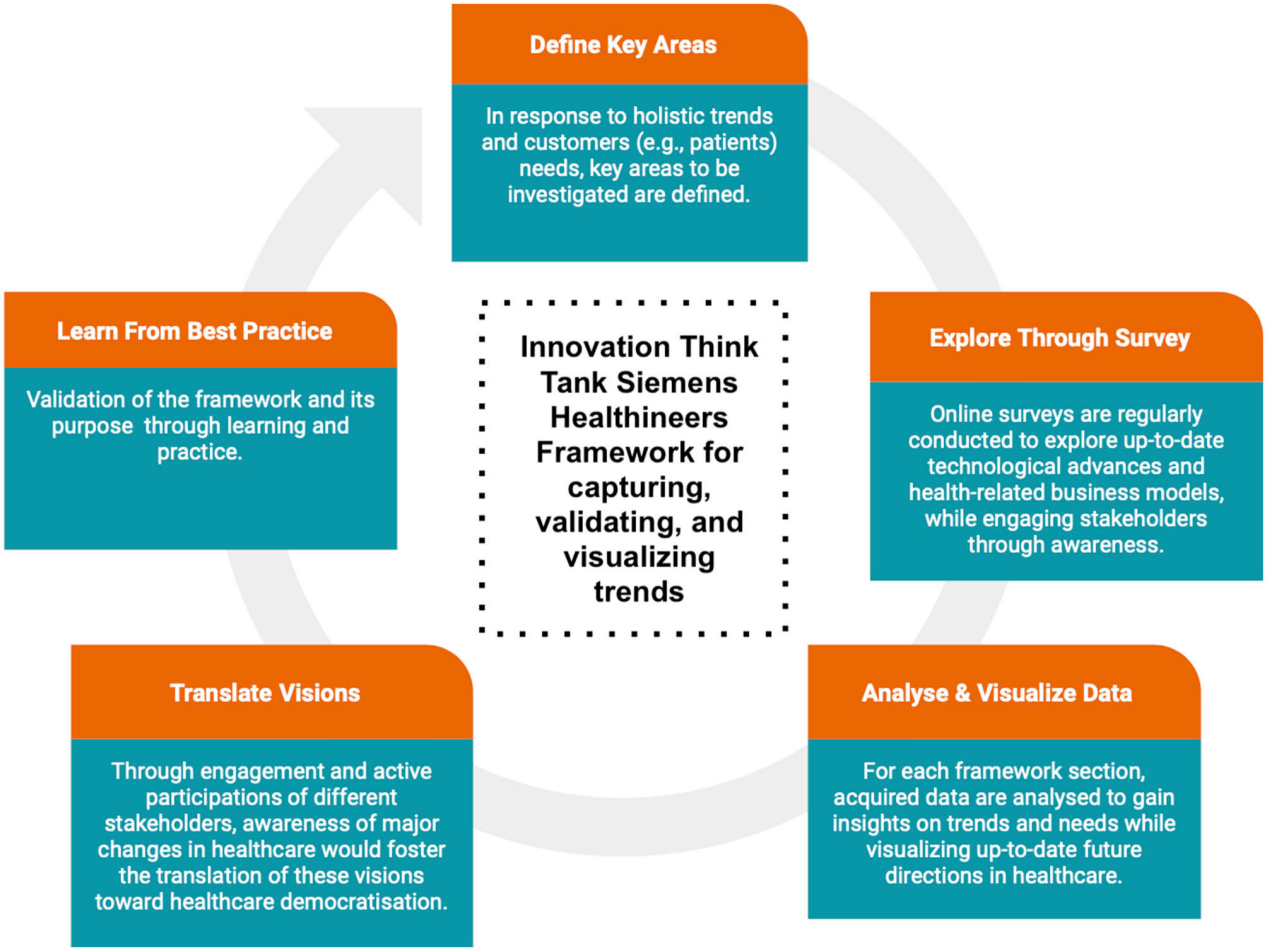
Innovation Think Tank Framework for capturing, validating and visualizing trends. In the first phase, areas to be further investigated are identified. This phase concerns the detection of future holistic trends and customers' needs. In the second phase, online surveys are regularly conducted to explore up-to-date advances regarding medical technologies and health-related business models to promote novel value propositions. In this phase, stakeholders are engaged through awareness and proactive participation. The third phase concerns the analysis and visualization of collected data. Thus, the investigation of data would allow insight into future trends and needs based on which healthcare should focus. In the fourth phase, future visions are translated into actions, in which different stakeholders are engaged toward the promotion of change and healthcare democratization. Lastly, in the fifth phase, the developed framework and methodology are constantly evaluated and validated based on learning and practice.

The developed framework (see Figure 1) was created around five sections to investigate relevant key areas in which healthcare innovation should focus. Moreover, from the structure of analyzed data, a visual inspection of holistic trends and interdependencies would be possible, with the goal of preparing healthcare professionals but also the global population regarding the future of healthcare (e.g., which are the primary needs and problems to be solved).

The first section “Demographics,” was aimed to collect demographic and personal information. The second section “Disruption in Health Technologies” was aimed at investigating the opinion of respondents in adopting advanced technologies to improve the healthcare process in a short and long-term perspective. The third section “Mutually reinforcing technologies,” was aimed at identifying which technologies will mutually reinforce each other to generate faster innovation and with that to provide disruption in healthcare opportunities. The fourth section “Novel value proposition to generate innovative healthcare” was aimed at considering respondents' opinions about how to add innovation to the current healthcare business model while generating novel value propositions. Finally, the last section “Forecasting the future of healthcare,” was aimed at exploring predictions about the future of healthcare, its advantages, and downsides, particularly how these changes would impact its structure and business model.

This framework represented the methodology, by which, Siemens Healthineers Innovation Think Tank investigates stakeholders' opinions about future holistic trends in healthcare while engaging them through awareness and active participation. Thus, the developed framework is translated into an online survey that would be regularly conducted to detect up-to-date technological advances, health-related business models, and interdependencies of innovative care deliveries and clinical needs.

In particular, our purpose was to:

i. Understand future trends and customers (e.g., patients) needs through engagement;
ii. Get information on future needs to improve and adapt the product portfolio;
iii. Regularly conduct investigations on up-to-date outcomes;
iv. Stimulate awareness of clinical needs and services toward healthcare democratization;
v. Learn from the innovation best practice.

Thus, to explore the future of health and needed innovations, an online survey was conducted among the medical staff of different departments and organizations. We designed the online survey using GOOGLE Forms. The survey consisted of 21 questions subdivided for each section of the above-mentioned framework. The questions were presented in the format of multiple-choice, checkboxes, five-point Likert scale, drop-down lists, and short- and long-answers text. Eighty-one responses were collected in total. Survey questions and answers are listed in Appendix A (Supplementary Material).

The survey answers were statistically analyzed based on the frequency distributions. The frequencies were computed based on the median distribution. In particular, the most frequent answers were transformed into their valid percentage.

## Result

From the survey analysis, the majority of respondents were male (69.1%) in the age of 25–34 (34.6%) and 18–24 (30.9%) years old, coming from Germany (39.5%) and India (16%), mostly working in the MedTec R&D (27.2%), innovation management (30.9%), and other sectors (29.6%) such as medical engineering, academic genetic research, innovation, engineering, healthcare technologies, and employees from the companies innovation lab from global locations (20).

**Figure 2:**
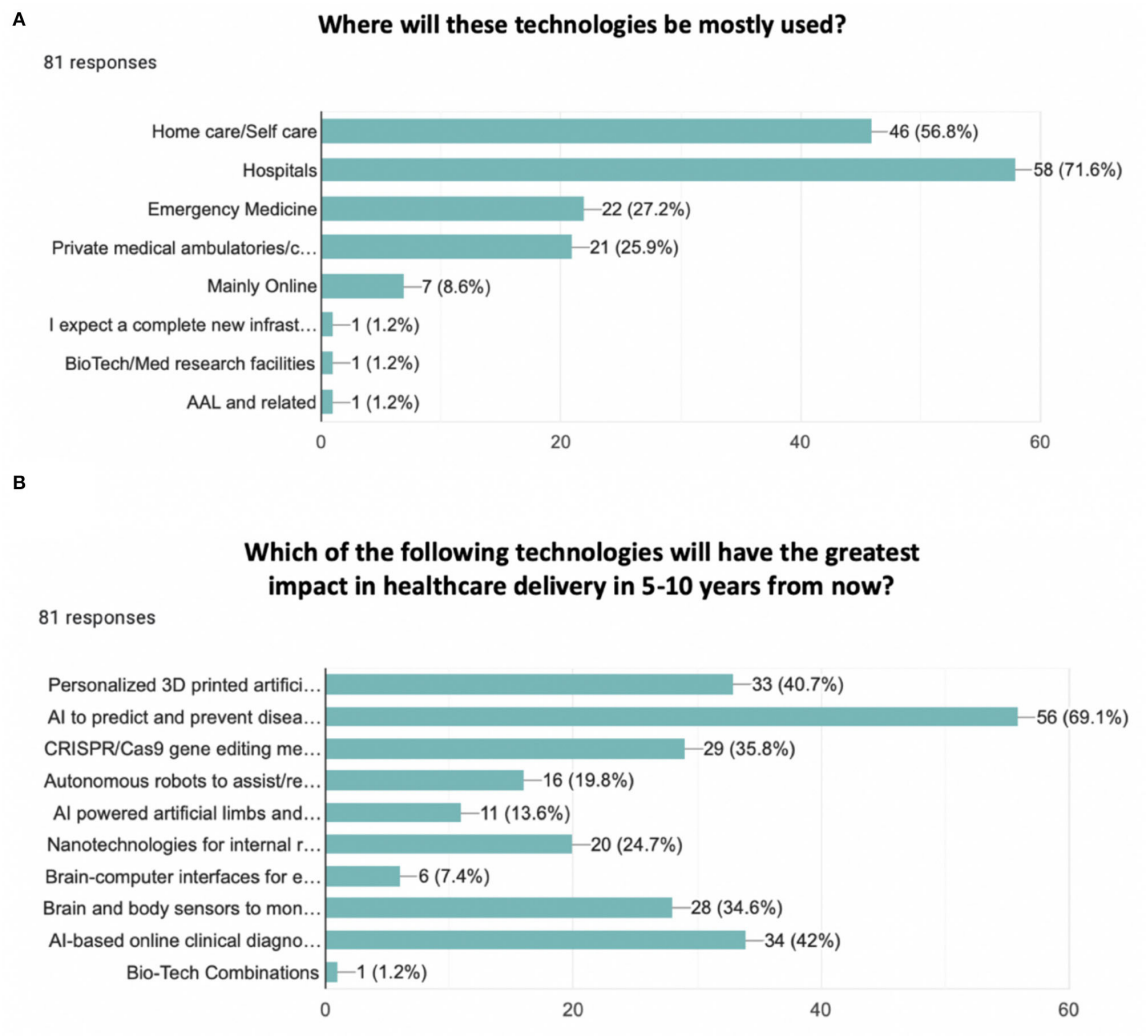
Bar chart. **(A)** Where will these technologies be mostly used? Options were: Home care/self-care, hospitals, emergency medicine, private medical ambulatories/centers, mainly online, others. The most two relevant responses were hospitals (71.6%) and home care/self-care (56.8%). **(B)** Which of the following technologies will have the greatest impact in healthcare delivery in 5–10 years from now? Options were: personalized 3D printed artificial organs/limb prosthetics/skin, AI to predict and prevent disease development, CRISPR/Cas9 gene editing method to treat incurable diseases (e.g., HIV, cancer, malaria, Huntington's disease, dementia), autonomous robots to assist/replace surgeons, AI-powered artificial limbs and exoskeletons, Nanotechnologies for internal repair and medication, Brain-computer interfaces for enhanced rehabilitation, brain and body sensors to monitor in real-time physiological and cognitive functioning, AI-based online clinical diagnostics, Other. The most three common answers were AI to predict and prevent disease development (69.1%), personalized 3D printed artificial organs/limb prosthetics/skin (40.7%), and AI-based online clinical diagnostics (42%).

**Figure 3:**
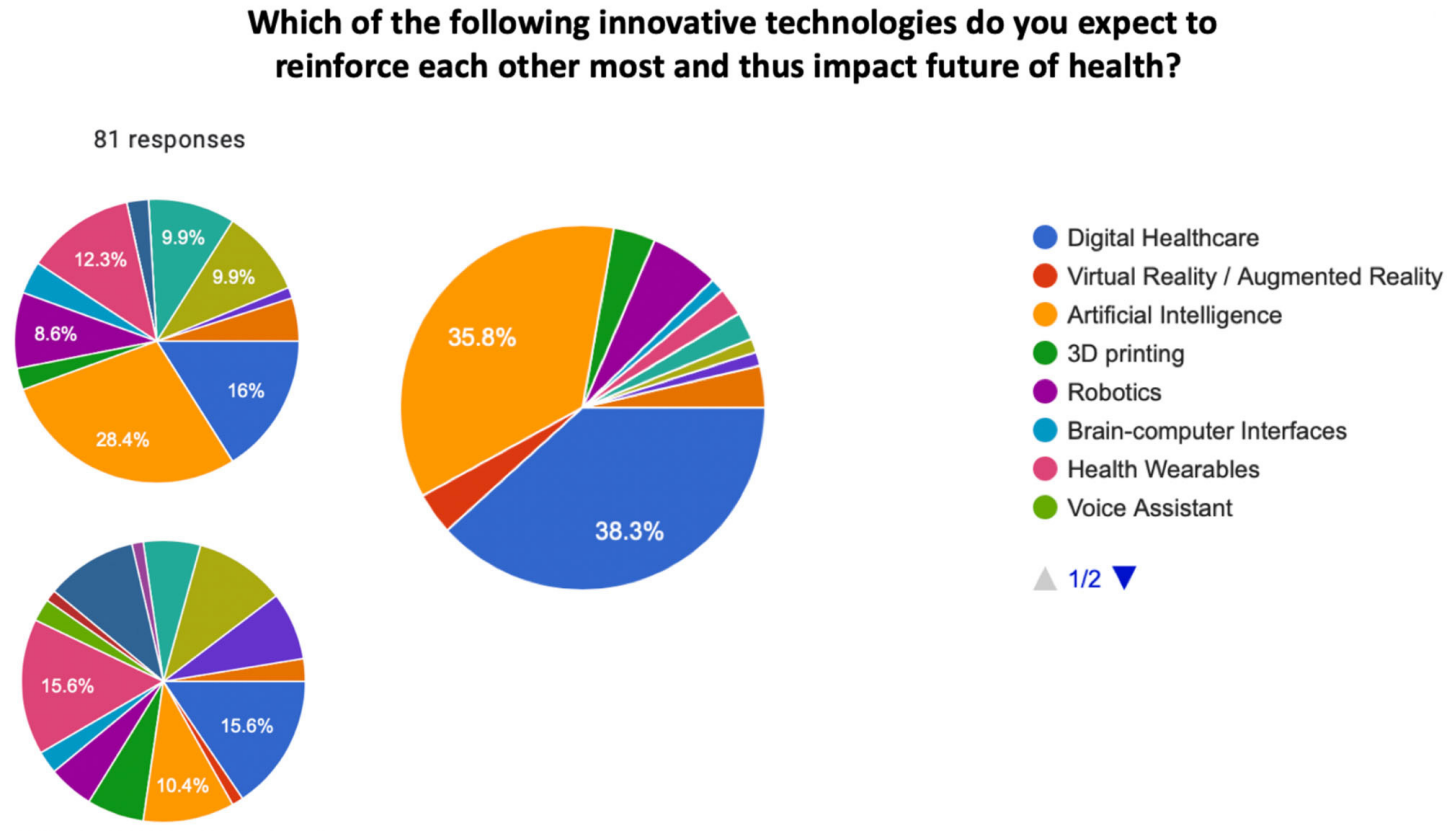
Pie chart. Which of the following innovative technologies do you expect to reinforce each other most and thus impact the future of health? Options were: digital Healthcare, virtual reality / augmented reality, artificial intelligence, 3D printing, robotics, Brain-Computer Interfaces, health wearables, voice assistant, new touch interfaces, minimal invasive therapy systems, environmental protection and sustainability, data management, electronic and sensors, cybersecurity and data privacy, gene editing (CRISPR/Cas9). The most selected technologies to mutually reinforce each other were artificial intelligence, digital healthcare, and health wearables.

When investigating which innovative technologies would mostly be expected to be implemented to improve the future of health in a 3–5 years perspective, the most three relevant responses were not surprisingly digital healthcare, electronic and sensors, and data management. However, the same technologies were also the most selected when forecasting the future of health in a 10 years perspective. Moreover, hospitals (71.6%) and home care/self-care (56.8%) were considered the two main places in which these technologies are expected to be adopted (see Figure 2A). While exploring healthcare delivery and procedures in the next 5–10 years, AI to predict and prevent disease development (69.1%), personalized 3D printed artificial organs/limb prosthetics/skin (40.7%), and AI-based online clinical diagnostics (42%) were identified as the main three technologies that would have the greatest impact on healthcare delivery (see Figure 2B). Furthermore, when exploring which innovative technologies would mostly reinforce each other to impact the future of health, AI, digital healthcare, and health wearables were considered the most impactful combination (see Figure 3).

We additionally investigated the sectors in which healthcare research should put more effort into generating innovative value propositions. While the most three common answers were digital healthcare, increased quality of healthcare services, the democratization of healthcare services, and increased user experience of the delivered services, other factors such as preventive/predictive measures, and faster accessibility of healthcare services for patients were identified as the most three important factors for the future of health. Moreover, a patient-centric ecosystem (i.e., predictive, preventive, personalized, participatory medicine), digitalization to enable faster and more efficient service delivery and operations (e.g., digital ecosystem), and the shift in perspective from a reactive to proactive and predictive healthcare system were selected the most three impactful value propositions to generate innovation.

**Figure 4:**
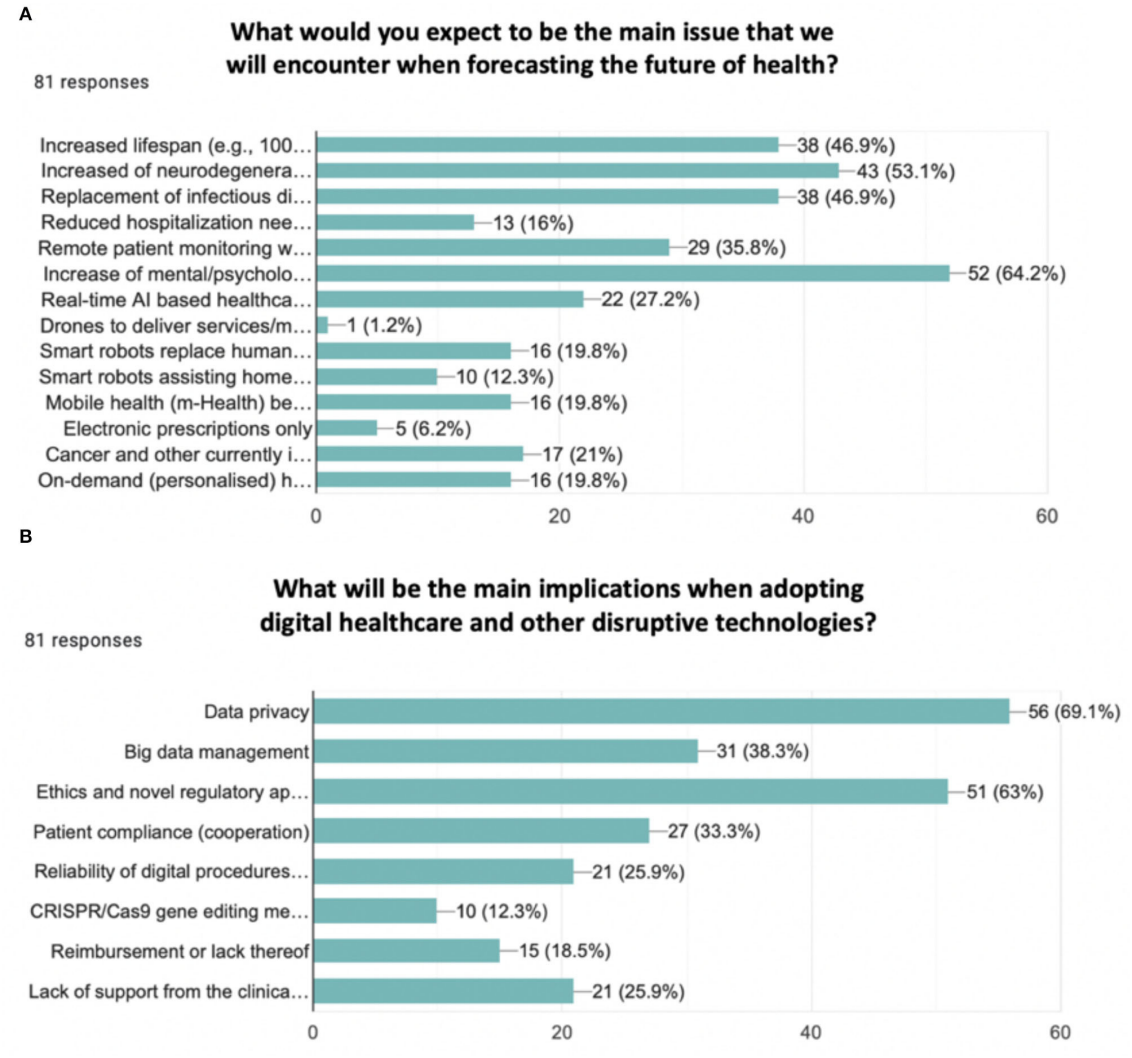
Bar chart. **(A)** What would you expect to be the main issue that we will encounter when forecasting the future of health? Options were: increased lifespan (e.g., 100 years of life perspective), increased of neurodegenerative disorders (e.g., Alzheimer's disease and other dementias), replacement of infectious diseases with increased chronic diseases (e.g., cardiovascular, pulmonary disease, type 2 diabetes, osteoporosis, arthritis, cancer), reduced hospitalization needs and nursing assistance, remote patient monitoring with digital healthcare systems (e.g., visiting the doctor in person will become a privilege), increase of mental/psychological illnesses, real-time AI-based healthcare home assistance, drones to deliver services/medical products (e.g., especially in undeveloped areas), smart robots replace humans at the hospital/pharmacy/medical ambulatory reception, smart robots assisting home care, mobile health (m-Health) becomes the standard of operations/procedures/services, electronic prescriptions only, cancer and other currently incurable diseases can be treated with pills or injections, on-demand (personalized) healthcare services. The most five selected responses were: increased lifespan (46.9%), increased of neurodegenerative disorders (53.1%), replacement of infectious diseases with increased chronic diseases (46.9%), remote patient monitoring with digital healthcare systems (35.8%), and increase of mental/psychological illnesses (64.2%). **(B)** What will be the main implications when adopting digital healthcare and other disruptive technologies? Options were: data privacy, big data management, ethics, and novel regulatory approvals, patient compliance (cooperation), reliability of digital procedures and clouds (e.g., to acquire, process, store, and exchange data), CRISPR/Cas9 gene-editing method, reimbursement or lack thereof, lack of support from the clinical profession. The most three selected responses were data privacy (69.1%), big data management (38.3%), and ethics and novel regulatory approvals (63%).

While forecasting the future of health, exploring the opinion of respondents about who should be in charge of paying for the implementation of technologies, the combination of insurance and government (61.7%) was the most common answer. Moreover, investigating which would be the main issue that will be encountered in the future, increased lifespan (e.g., 100+ years of healthy longevity) (46.9%), increases of neurodegenerative disorders (e.g., Alzheimer's disease and other dementias) (53.1%), replacement of infectious diseases with chronic diseases (e.g., cardiovascular, pulmonary diseases, type 2 diabetes, osteoporosis, arthritis, cancer) (46.9%), remote patient monitoring with digital healthcare systems (e.g., visiting the doctor in person will become a privilege) (35.8%), and an increase of mental/psychological illnesses (64.2%) were the most relevant answers (see Figure 4A). When speculating about the main implications of adopting digital healthcare and other disruptive technologies, data privacy (69.1%), big data management (38.8%), ethics, and novel regulatory approvals (63%) were the most often provided answers (see Figure 4B).

In addition, novel and future-oriented healthcare teaching and education programs were considered important (23.5%) very important to generate innovation in healthcare (66.7%). Likewise, the consideration of introducing innovative labs exploring disruptions besides the already ongoing core incremental innovation activities were considered important (27.2%) or very important (54.3%). Intentional disruption was also considered important (35.8%) or very important (44.4%) in the future of respondents' organizations and activities.

Lastly, respondents were asked to share their opinion about values and perspectives considered important to promote innovation in healthcare. The most common and relevant thoughts were related to the need of empowering patients with transparent data to support their health journey. For the P4 of health (i.e., preventing, predicting, personalized and participatory medicine) to become reality it is also necessary to embrace disruption while promoting health innovation, moving from a reactive to a preventive and proactive care system, and a novel health business model that is outcome-based oriented. Nevertheless, awareness, knowledge, and acceptance of advanced medical technologies become also a public health concern that should be supported across health professionals, and in the general population. The shift toward an innovative mindset would also impact the way of training and educating future clinicians and healthcare professionals. Hence, a revised educational program should be proposed to accomplish the acquisitions of multiple skills into an interdisciplinary setting, and where clinical research merges with biomedical engineering toward a perspective of a novel healthcare ecosystem.

## Discussion

Healthcare is a system characterized by a complex structure in which different stakeholders come into play, such as care providers, insurers, policymakers, and patients interacting in a dynamic and synergic way. However, the current healthcare system suffers from a few burdens that limit the operation of the entire organization, care delivery, and management, and in which the lack of patient safety, quality care, unsustainable costs, and reactive measures raise the need for innovation. Thus, the healthcare system is forced to accelerate its transformation toward the establishment of novel value propositions, and in which the healthcare organization is re-designed shifting the perspective from hospital-based to social- and homecare-based systems (21). Hence, large-scale transformations should be encouraged to evolve this system overcoming the current obstacles.

Moreover, the need to evolve clinical practice into a transformed ecosystem is also the response to 21st-century challenges (11). Indeed, Industry 4.0 and digital transformation brought disruptive and innovative technologies to enter our workplaces and private life and thus influencing the healthcare system to digitally transform. However, even though healthcare is not yet ready to shift toward a digitalized and decentralized system, the Covid-19 pandemic represented a considerable event while forcing the urgency of remote E-health services and monitoring. Whether it is clear that digitalization and advanced technologies in healthcare would bring opportunities and a novel set of values (17), the importance of revising education and training programs cannot be neglected to allow this transformation (14). Moreover, learning how to implement these advances to enhance patient care and experience (18) is the key when thinking of which aspect and innovative strategy should healthcare invest its effort in.

In this regard, our study was firstly aimed to investigate the future of health from a technological and clinical point of view. Secondly, our ultimate goal is the realization of these visions to improve services, procedures, and health democratization in a perspective that is patient-centric, preventive, predictive, and proactive (15, 19). We developed a framework to detect relevant areas of investigation, followed by the analysis and visualization of holistic trends concerning advanced medical technologies and innovative health-related business models. Indeed, the methodology would help the visualization and consequent translation of predicted technological and clinical developments toward healthcare democratization. Thus, we engaged stakeholders by conducting an online survey to explore the opinion of participants, working in different medical sectors, about the future of healthcare in a short term (3–5 years) and long term (>10 years) perspective.

Digital healthcare, electronic and sensors, and data management have been found to be the three most innovative technologies that would be implemented in healthcare in a short and long-term perspective. Not surprisingly, the future of healthcare is already moving toward the implementation of digital platforms, and globally connected networks while generating a novel ecosystem that is dynamic, autonomous, and made of multiple decentralized stakeholders. Moreover, hospitals and home care were considered the two main settings where advanced technologies and procedures are mostly expected to be implemented. Thus, the future of health will rotate around two main protagonists such as hospitals and homes, and in which the majority of procedures will take place. According to Zimlichman et al. (21) hospital-at-home programs would improve patients' outcomes with increased satisfaction at lower costs. This trend would also allow a personalized approach to health preventing and predicting the occurrence of diseases besides reducing long hospitalizations. Thus, this will represent what is called telehealth; a decentralized system, remotely accessible, and able to guarantee real-time monitoring, while shifting care services from hospitals to homes.

Moreover, to enhance health delivery in a 5–10 years perspective, AI systems stand out among other advanced technologies. Indeed, in clinical diagnostic, AI can predict and prevent disease development, while supporting physicians in making better decisions suitable for specific patients' needs. Besides, by combining AI with health wearables, patients monitoring devices will be the solution for continuous remote health monitoring and digital-based services.

When approaching innovation, a novel set of values should be established to transform the hospital-based delivery model into a community, home-based care system. While allowing faster health accessibility, prevention, prediction, and a user-based approach digital healthcare has been found to be the core value on which healthcare should focus. At the same time, the democratization of services through telemedicine would create a patient-centric and digital ecosystem (i.e., the P4 of medicine) to elevate efficiency and efficacy. Thus, digital technologies become the essential tools, in which a different understanding of healthcare delivery is going to move the attention from sickness to health, and from reaction to prevention (15, 19). However, becoming telemedicine and digital care the primary drivers of healthcare disruption, the importance of transparency, integrity, openness, clear semantics, and interoperability is essential (22).

Furthermore, although promoting innovation has become a necessity, the importance of identifying who is responsible for financially supporting the change should also be considered. Hence, participants reported that a combination of insurance and government should be in charge of this process. In addition, even payment systems are going to change, transforming from reimbursement to value-based models. The outcome is health, and what any providers should incentivize is again health. Hence, promoting a novel culture in which providers and payers encourage the community to actively participate in health education, would have a considerable impact on preventing disease development, as well as, a positive impact on socioeconomic conditions (21). Accordingly, forecasting the future of healthcare (19) means also the capability to predict which would be the main encountered issues. Indeed, their understanding would facilitate the way healthcare will cope with them.

Among these, increased lifespan, increased neurodegenerative disorders, increased chronic diseases, digital and remote patient monitoring, and an increase of mental/psychological illnesses have been recognized as the most relevant issue that healthcare will encounter in a few years. Besides data privacy, big data management, ethics, and novel regulatory approvals were identified as the main implications to be faced when implementing digitization and advanced technologies in healthcare. Thus, when approaching digital transformation in healthcare delivery the need for revised education and training programs is necessary when preparing future professionals for innovation generation (14). Indeed, participants agreed that this factor should be considered, as well as, the promotion of activities, classes, and labs to conduct innovative initiatives. This would translate into a context that is interdisciplinary and in which research, clinical, and biomedical engineering co-create to build the future of health in a dynamic and complex system.

**Figure 5:**
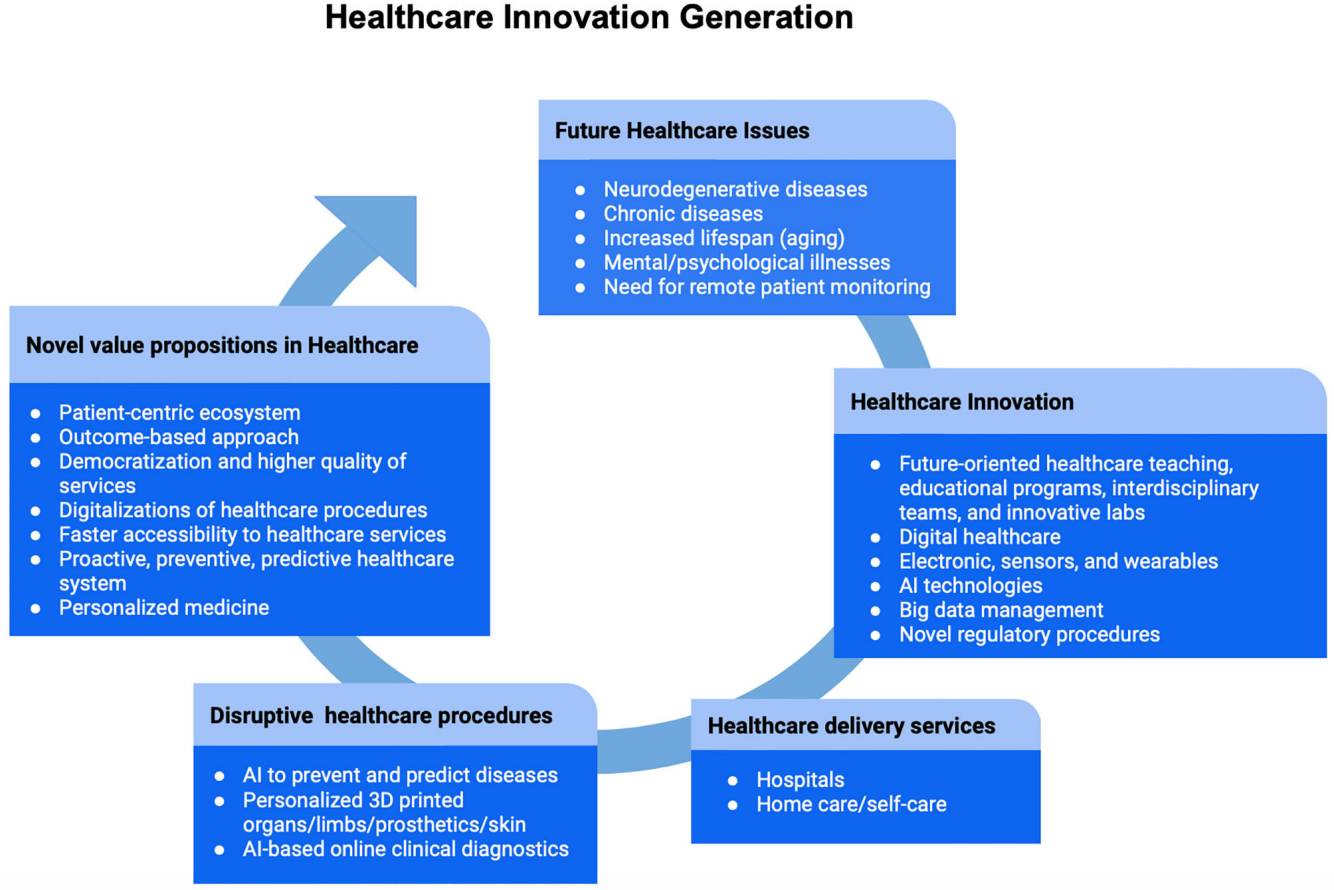
Healthcare innovation generation. A summary of the main results is represented in the diagram. Future healthcare issues will be mostly determined by increased neurodegenerative and chronic diseases, followed by demographic changes characterized by the aging population, mental/psychological illnesses, and need for remote patient monitoring. In response to these challenges, the entire healthcare system needs to embrace innovation. Therefore, the process of innovation will be characterized by a revised education to prepare future healthcare professionals, digital services, novel regulatory procedures, a better management of big data, and the implementation of advanced medical technologies. In particular, hospitals and home care/self-care are the two main places where innovative healthcare delivery services will take place. Furthermore, the implementation of disruptive technologies will empower healthcare with preventive, predictive, and personalized procedures. Generating novel value propositions means the promotion of a proactive system, focused on health and prevention. With a patient-centric and outcome-based approach, followed by the democratization and faster accessibility of care services through digitalization, healthcare will be capable of embracing innovation while facing the rapid changes brought by the 21^st^-century.

In conclusion, based on these survey results, a deep dive and business impact assessments for promising fields will further be conducted. Indeed, our developed framework would represent a model to regularly conduct online surveys while investigating up-to-date technological advances, health-related business models, and interdependencies in healthcare. To realize these visions (see Figure 5; Table 1), the future of healthcare is going to evolve toward a system in which a patient-centric and value-based based approach is fostered through empathy and where preventive and predictive measures would help the understanding of a disease-pathway strategy, consequently improving life quality and daily functioning (23, 24).

**Table 1:** Healthcare visions based on the survey results.

**Current healthcare services**	**Key learnings from the study with respect to the future of health**
Hospital-based care	Shift to Home-based care
Focus on treatments	Focus on prevention and prediction
Reimbursement-based	Outcome-based
Long hospitalizations and generalized patient's management and monitoring	Remote patient monitoring with a personalized care approach
Insurance-based care services	Democratization of services based on telemedicine
Traditional clinical education and training	Interdisciplinary teams made of clinical and engineering experts
Generalized treatments	Precision medicine and disease-pathway strategies

## Conclusion

Digital transformation characterizing our century forced manufacturers and tech industries to adapt their business model while dealing with upcoming and foreseeable disruptions. Even the healthcare sector had to face this evolving trend coping with a series of challenges that affect this complex system globally. Thus, to survive disruption, the identification of novel value propositions and innovative solutions is needed. In this regard, a globally operating Medical Technology company constantly needs to investigate future technological and clinical developments in healthcare. Hence, conducting regular surveys among professionals is one means to get the needed insights.

With digital healthcare, remote sensors, and wearables, bringing care to homes becomes the main goal of healthcare delivery (24). Indeed, shifting services from a hospital-based to a home-based setting would transform this care delivery organization into a decentralized and digital ecosystem. Moreover, whether payment models become value-based, introducing AI technologies into clinics and diagnostic procedures has the advantage of preventing and predicting diseases, thus supporting healthcare providers to make more accurate decisions for the best patient outcome. Indeed, with a patient-centric approach, the importance of increased quality, democratization, and accessibility of services are important values to consider to ameliorate users' experience. However, forecasting the future of health means also identifying possible issues their acknowledgment would be useful when finding a proper solution. Due to aging and longevity, increased neurodegenerative and chronic diseases, as well as, increased mental, psychological illnesses, are expected to mostly occur. Besides, data privacy, big data management, and novel regulatory procedures are also implications to be faced when healthcare transforms into a digitalized ecosystem. Hence, revising education while preparing future professionals toward their mission in delivering high-quality care, must be considered when promoting an environment that is interdisciplinary and in which clinical subjects merge with medical engineering expertise.

With this study and developed framework, Innovation Think Tank Siemens Healthineers aims to consider this these visions and perspectives into their projects to foster and support the future of healthcare, as a democratized, digital, transparent, open, and multidisciplinary ecosystem (13–15, 19). Hence, an in-depth examination and business impact assessment will be regularly conducted to foresee up-to-date exponential technological, innovative business models, and interdependencies. Thus, engaging stakeholders through awareness and proactive participation will be the key to understanding clinical needs and related novel care deliveries, toward the perspective of healthcare democratization.

## Data Availability Statement

The raw data supporting the conclusions of this article will be made available by the authors, without undue reservation.

## Ethics Statement

Ethical review and approval was not required for the study on human participants in accordance with the local legislation and institutional requirements. Written informed consent for participation was not required for this study in accordance with the national legislation and the institutional requirements.

## Author Contributions

BB designed the survey, carried out the experiment, and wrote the manuscript with support and revision from MF and SH. MF and SH supported the project with respect to Siemens Healthineers, conceived the original idea, and supervised the project. All authors contributed to the article and approved the submitted version.

## Funding

The project has been financially supported by Siemens Healthineers.

## Conflict of Interest

This study received funding from Siemens Healthineers. The funder had the following involvement with the study: study design, data collection, project supervision, revision of the manuscript, and decision to submit the manuscript for publication. The authors declare that the research was conducted in the absence of any commercial or financial relationships that could be construed as a potential conflict of interest.

## Publisher's Note

All claims expressed in this article are solely those of the authors and do not necessarily represent those of their affiliated organizations, or those of the publisher, the editors and the reviewers. Any product that may be evaluated in this article, or claim that may be made by its manufacturer, is not guaranteed or endorsed by the publisher.
