# Supplementary material for: Engaging Through Awareness: Purpose-Driven Framework Development to Evaluate and Develop Future Business Strategies With Exponential Technologies Toward Healthcare Democratization
Source: Front Public Health. 2022 May 25;10:851380. doi: 10.3389/fpubh.2022.851380 (PMC9174566; doi:10.3389/fpubh.2022.851380)
Supplement: Supplementary file 1 [file Table_1.docx]

Supplementary Material

# Supplementary Figures and Tables

The survey questions and answers are recorded in the following supplementary files.

**Appendix A**

| **Siemens Healthineers - Survey Questions** |
| --- |
| 1. **1. Demographics** |
| 1. Sex 2. Age 3. Please, select your country 4. Education/job sector - if other, please specify |
| **2. Disruption in Health Technologies** |
| 1. Which of the following INNOVATIVE TECHNOLOGIES do you expect to be implemented in a 3-5 years horizon to improve FUTURE HEALTH? Rate 1-3 (where 1=not really implemented, 2=maybe, 3=mostly implemented) 2. Which of the following INNOVATIVE TECHNOLOGIES do you expect to be implemented in 10 years from now to improve FUTURE HEALTH? Rate 1-3 (where 1=not really implemented, 2=maybe, 3=mostly implemented) 3. Where will these technologies be mostly used? (Select the 2 most likely options) 4. Which of the following technologies will have the greatest impact on healthcare delivery in 5-10 years from now? (Select the 3 most likely options) |
| **3. Mutually Reinforcing Technologies** |
| Which of the following INNOVATIVE TECHNOLOGIES do you expect to reinforce each other most and thus impact FUTURE HEALTH? For example, if you expect that Digital Healthcare, AI, and Robotics will reinforce each other and their combination will heavily impact FUTURE HEALTH, select them one at a time in each drop-down menu (e.g., a combination of 3 technologies). If you expect only a combination of Robotics and Brain-computer interfaces to reinforce one another, select them one at a time in each of the first two drop-down menus (e.g., a combination of 2 technologies). You can select up to three mutually reinforcing technologies.     1. Please, select one technology to create up to three mutually reinforcing technology combinations (1) 2. Please, select one technology to create up to three mutually reinforcing technology combinations (2) 3. Please, select one technology to create up to three mutually reinforcing technology combinations (3) |
| **4. Novel Value Proposition To Generate Innovative Healthcare** |
| 1. In which segment should we put our development efforts? Rate your answer 1-5 (where 1=not at all needed, 3=neutral, 5=very needed) 2. What do you see as the most important aspect of the FUTURE of HEALTH? Rate your answer 1-5 (where 1=not very important, 3=neutral, 5=very important) 3. What would be the most IMPACTFUL value proposition for future healthcare innovation? Rate your answer 1-5 (where 1=not very impactful, 3=neutral, 5=very impactful) |
| **5. Forecasting The Future Of Healthcare** |
| 1. In your opinion, who should be in charge of paying for the implementation of these advanced technologies? 2. What would you expect to be the main ISSUE that we will encounter when forecasting the future of health? (Select the 5 most likely options) 3. What will be the main implications when adopting digital healthcare and other disruptive technologies? (Select the 3 most likely options) 4. How important are novel and future-oriented teaching and education programs dealing with innovation generation in healthcare? Rate your answer 1-5 (where 1=not very important, 3=neutral, 5=very important) 5. How important do you consider an innovation lab in your organization that deals exclusively with disruptions (EDGE) besides the ongoing CORE innovation activities? Rate your answer 1-5 (where 1=not very important, 3=neutral, 5=very important) 6. How important do you believe will INTENTIONAL DISRUPTION directly or indirectly be for your organization in the future? Rate your answer 1-5 (where 1=not very important, 3=neutral, 5=very important) 7. Which value/aspect would you believe important to consider toward promoting healthcare innovation? (e.g., I believe that...) |

| **Siemens Healthineers - Survey Answers** |
| --- |
| **1. Demographics** |
| ●      18-24  ●      25-34  ●      35-50  ●      >50 |
| ●      Male  ●      Female  ●      Diverse |
| - Afghanistan - … - … - … - … - Zimbabwe |
| - Pharma R&D - MedTec R&D - Clinician Hospital - Clinician Ambulatory Care - Health Administration - Project Management - Innovation Management - Health Services - Other |
| Short-answer text |
| **2. Disruption in Health Technologies** |
| - Digital Healthcare - Virtual Reality / Augmented Reality - Artificial Intelligence - 3D Printing - Robotics - Brain-Computer Interfaces - Health Wearables - Voice Assistant - New Touch Interfaces - Minimal Invasive Therapy Systems - Environmental Protection and Sustainability - Data Management - Electronic and Sensors - Cybersecurity and data privacy - Gene editing (CRISPR/Cas9) |
| - Digital Healthcare - Virtual Reality / Augmented Reality - Artificial Intelligence - 3D Printing - Robotics - Brain-Computer Interfaces - Health Wearables - Voice Assistant - New Touch Interfaces - Minimal Invasive Therapy Systems - Environmental Protection and Sustainability - Data Management - Electronic and Sensors - Cybersecurity and data privacy - Gene editing (CRISPR/Cas9) |
| - Home care/Self-care - Hospitals - Emergency Medicine - Private medical ambulatories/centers - Mainly Online - Other |
| - Personalized 3D printed artificial organs/limb prosthetics/skin - AI to predict and prevent disease development - CRISPR/Cas9 gene editing method to treat incurable diseases (e.g., HIV, cancer, malaria, Huntington's disease, dementia) - Autonomous robots to assist/replace surgeons - AI-powered artificial limbs and exoskeletons - Nanotechnologies for internal repair and medication - Brain-computer interfaces for enhanced rehabilitation - Brain and body sensors to monitor in real-time physiological and cognitive functioning - AI-based online clinical diagnostics - Other |
| **3. Mutually Reinforcing Technologies** |
| - Digital Healthcare - Virtual Reality / Augmented Reality - Artificial Intelligence - 3D Printing - Robotics - Brain-Computer Interfaces - Health Wearables - Voice Assistant - New Touch Interfaces - Minimal Invasive Therapy Systems - Environmental Protection and Sustainability - Data Management - Electronic and Sensors - Cybersecurity and data privacy - Gene editing (CRISPR/Cas9) |
| - Digital Healthcare - Virtual Reality / Augmented Reality - Artificial Intelligence - 3D Printing - Robotics - Brain-Computer Interfaces - Health Wearables - Voice Assistant - New Touch Interfaces - Minimal Invasive Therapy Systems - Environmental Protection and Sustainability - Data Management - Electronic and Sensors - Cybersecurity and data privacy - Gene editing (CRISPR/Cas9) |
| - Digital Healthcare - Virtual Reality / Augmented Reality - Artificial Intelligence - 3D Printing - Robotics - Brain-Computer Interfaces - Health Wearables - Voice Assistant - New Touch Interfaces - Minimal Invasive Therapy Systems - Environmental Protection and Sustainability - Data Management - Electronic and Sensors - Cybersecurity and data privacy - Gene editing (CRISPR/Cas9) |
| **4. Novel Value Proposition To Generate Innovative Healthcare** |
| - Digital healthcare - Availability of services and tools online - Reduction of service costs - Democratization of healthcare services - Increased quality of healthcare services - Increased user experience of services - Revised healthcare education |
| - Democratization of services access (eliminate inequalities) - Cost reduction of health-related services (affordability) - Improve patient experience - Preventive measures - Predictive measures - Proactive and engaging participation - Increased quality of healthcare services - Faster diagnostics - Availability of health data for patients - Telehealth services - Faster accessibility of healthcare services for patients |
| - Patient-centric ecosystem (i.e., predictive, preventive, personalized, participatory medicine) - Healthcare as a complex, self-organized, and dynamic system (e.g., non-linear interactions between patient, caregivers, hospital units, government) - Patient empowerment (e.g., active collaboration with providers becoming aware of their own care) - Reduced healthcare costs and more affordable services (e.g., from providers, insurance, and suppliers) - Value-based on outcomes (e.g., differently from reimbursement-based value) - e-Health to guarantee complete, accurate documentation and billing information - Continuous monitoring of patient's health (e.g., digital databases based-AI) - Telehealth to reach a remote environment - Blockchain electronic health records - Digitalization to enable faster  and more efficient service delivery and operations (e.g., digital ecosystem) - Empathy to value patients' journey (e.g., data sharing, empowerment of medical decisions, continuous supervision) - Healthcare from reactive to proactive and predictive care process |
| **5. Forecasting The Future Of Healthcare** |
| - Health Insurance - Government of your country - Self-payers - Combination of insurance and self-payer - Combination of insurance and government - Combination of government and self-payer - Other |
| - Increased lifespan (e.g., 100 years of life perspective) - Increased of neurodegenerative disorders (e.g., Alzheimer's disease and other dementias) - Replacement of infectious diseases with increased chronic diseases (e.g., cardiovascular, pulmonary disease, type 2 diabetes, osteoporosis, arthritis, cancer...) - Reduced hospitalization needs and nursing assistance - Remote patient monitoring with digital healthcare systems (e.g., visiting the doctor in person will become a privilege) - Increase of mental/psychological illnesses - Real-time AI-based healthcare home assistance - Drones to deliver services/medical products (e.g., especially in undeveloped areas) - Smart robots replace humans at the hospital/pharmacy/medical ambulatory reception - Smart robots assisting home care - Mobile health (m-Health) becomes the standard of operations/procedures/services - Electronic prescriptions only - Cancer and other currently incurable diseases can be treated with pills or injections - On-demand (personalized) healthcare services |
| - Data privacy - Big data management - Ethics and novel regulatory approvals - Patient compliance (cooperation) - Reliability of digital procedures and clouds (e.g., to acquire, process, store, and exchange data) - CRISPR/Cas9 gene editing method - Reimbursement or lack thereof - Lack of support from the clinical profession |
| 5-points Likert scale |
| 5-points Likert scale |
| 5-points Likert scale |
| Long-answer text |
